# High Thymic Output of Effector CD4^+^ Cells May Lead to a Treg : T Effector Imbalance in the Periphery in NOD Mice

**DOI:** 10.1155/2019/8785263

**Published:** 2019-06-11

**Authors:** Yuan Zhao, Pascale Alard, Michele M. Kosiewicz

**Affiliations:** ^1^Department of Microbiology and Immunology, University of Louisville Health Sciences Center, Louisville, KY 40202, USA; ^2^Department of Pharmaceutical Sciences, Sullivan University College of Pharmacy and Health Sciences, 2100 Gardiner Lane, Louisville, KY 40205, USA

## Abstract

Regulatory T cells (Tregs) play a critical role in controlling autoreactive T cells, and quantitative and/or qualitative deficiencies in Tregs are associated with autoimmune diseases, including type 1 diabetes (T1D), in both humans and mice. Both the incidence of T1D and percentages of peripheral Tregs in NOD mice vary considerably between animal facilities. In our animal facility, the incidence of T1D in NOD mice is high at 90-100% and the percentages of peripheral CD4^+^Foxp3^+^ cells in ~9-10-week-old female NOD mice are decreased compared to control (B6) mice shortly before high glucose is first detected (~12 weeks). These data suggest that there is an imbalance between Tregs and potentially pathogenic effector T cells at this age that could have significant impact on disease progression to overt diabetes. The goal of the current study was to investigate mechanisms that play a role in peripheral Treg : T effector cell balance in NOD mice, including differences in persistence/survival, peripheral homeostatic proliferation, and thymic production and output of CD4^+^ T cells. We found no differences in persistence/survival or homeostatic proliferation of either Tregs or effector T cells between NOD and B6 mice. Furthermore, although the percentages and absolute numbers of CD4^+^Foxp3^+^ cells in thymus were not decreased in NOD compared to B6 mice, the percentage of CD4^+^ recent thymic emigrants (RTE) that were Foxp3^+^ was significantly lower in 9-week-old NOD mice. Interestingly, the thymic output of CD4^+^Foxp3^+^ cells was not lower in NOD mice, whereas the thymic output of CD4^+^Foxp3^−^ cells was significantly higher in NOD mice at that age compared to B6 mice. These data suggest that the higher thymic output of CD4^+^Foxp3^−^ T cells contributes, at least in part, to the lower percentages of peripheral CD4^+^Foxp3^+^ Tregs in NOD mice and an imbalance between Tregs and T effector cells that may contribute to the development of full-blown diabetes.

## 1. Introduction

Regulatory T cells (Tregs) play a critical role in mediating peripheral tolerance by controlling autoreactive T cells. Depletion of CD4^+^CD25^+^ Tregs in animal models of autoimmune disease can exacerbate disease, and this can be overcome by reconstitution with this cell population. Animal and human studies suggest that Tregs play an important role in protection from type 1 diabetes (T1D). Whether it is the number and/or function of Tregs and/or the susceptibility of pathogenic T cells to suppression that are defective in T1D patients and the NOD mouse model of T1D is still controversial. Different laboratories have evaluated the percentages of CD4^+^CD25^+^ Tregs and have reported varying results [1–11]. Our earlier study indicated that the percentages of CD4^+^CD25^+^ cells were lower in NOD mice in our facility [1], and we also found differences in Foxp3 expression in Tregs between B6 and sick NOD mice [12]. Some of these earlier studies relied solely on CD25 as the marker for Tregs, while later studies utilized Foxp3. Consequently, some of the discrepancies in results could have been due to the differences in Treg markers. The differences in the results in more recent studies that use Foxp3 as a marker may be explained by the variation in animal facility environments. It is well-established that the incidence of T1D in NOD mice differs significantly between animal facilities. Although the average T1D incidence is ~80% in female NOD mice, T1D incidence has been reported to range between 60 and 100% in different facilities and is heavily influenced by the “cleanliness” of the mouse colony and dietary factors, including food and water—all of which likely impact the microbiota [13–20].

Evidence suggests that an imbalance between Tregs and effector T cells may be a key determinant in the development of T1D [21, 22]. Therapies that augment the number of Tregs or restore the balance between Tregs and effector T cells have been reported to be critical in preserving islet *β*-cell function [21, 23–25]. Several clinical trials and murine studies including treatment with anti-CD3 monoclonal antibodies and leukocyte function antigen-3- (LFA-3-) Ig have demonstrated promising results in selectively depleting Teffs while preserving Tregs [26–28]. The low percentages of Tregs in the periphery of NOD mice in our colony suggest that there is an imbalance between the Tregs and potentially pathogenic Teffs and that this imbalance may directly affect the pathogenesis of T1D in these mice. The goal of the current project was to investigate the mechanisms that affect the Treg : Teff equilibrium in NOD mice in our facility. To this end, mechanisms involved in the maintenance of these CD4^+^ populations in the periphery were investigated. Mechanisms that can affect the maintenance of either Tregs or T effector cells, and therefore, the Treg to T effector ratio, in the periphery include the following: (1) cell persistence and/or survival, (2) peripheral homeostatic proliferation, and (3) thymic production and output.

In the current study, we have found a correlation between low percentages of Foxp3-expressing CD4^+^ Tregs in NOD mice and the high incidence of diabetes (90-100%) in our colony. Interestingly, the lower percentages of CD4^+^ Tregs in NOD mice are not due to differences in persistence, survival, or homeostatic proliferation of CD4^+^Foxp3^+^ or CD4^+^Foxp3^−^ cells in NOD compared to B6 (non-T1D-prone strain) mice but do seem to be due to a considerably higher rate of thymic export of CD4^+^Foxp3^−^ cells in NOD mice that was first observed shortly before disease onset (i.e., ~13-15 weeks) at 9-10 weeks of age. These data suggest that a higher thymic output of CD4^+^Foxp3^−^ T cells likely contributes, at least in part, to the low percentages of peripheral CD4^+^Foxp3^+^ Tregs (i.e., decreased Treg : Teff ratio) found in NOD mice in our colony, thus leading to an imbalance between Tregs and Teffs that may contribute to the development of the high incidence of full-blown diabetes.

## 2. Methods

### 2.1. Mice

Six-8-week-old female NOD/LtJ (NOD), NOD.NON-Thy1^a^/1LtJ (NOD.NON), C57BL/6 (B6), B6.SJL-Ptprc^a^ Pepc^b^/BoyJ (B6.SJL), B6.CB17-*Prkdc^scid^*/SzJ (B6.SCID), NOD.CB17-*Prkdc^scid^*/J (NOD.SCID), B6; 129S7-*Rag1^tm1Mom^*/J (B6.RAG), and NOD.129S7 (B6)-*Rag1^tm1Mom^*/J (NOD.RAG), were purchased from The Jackson Laboratory (Bar Harbor, ME). Mice were bred and maintained under specific pathogen-free conditions in the animal facility at the University of Louisville following the guidelines stipulated by the University of Louisville Institutional Animal Care and Use Committee.

### 2.2. Assessment of Diabetes

Female NOD mice were monitored weekly for glycosuria using Diastix (Bayer) beginning at 10 weeks of age. High glucose (~250 mg/dl) can first be detected in some mice at 12 weeks of age in our colony. Mice were considered diabetic only when they had two consecutive positive readings for high glucose (>250 mg/dl). The onset of diabetes was dated from the second positive readings of sequential measurements. Diabetes incidence is expressed as a percentage of mice with hyperglycemia. None of the mice at ≤12 weeks of age that were used in this study had full-blown diabetes.

### 2.3. Antibodies and Reagents

Commercially available monoclonal antibodies used in this study were as follows: FITC or PerCP anti-CD4; FITC or APC anti-CD8; APC or PE anti-CD25; FITC anti-CD45.1, anti-CD45.2, anti-Thy1.2, and anti-BrdU; and anti-CD3 (2C11) mAbs (BD Pharmingen, CA). PE or APC anti-Foxp3 antibodies were purchased from eBioscience (CA). Carboxyfluorescein diacetate succinimidyl ester (CSFE) was purchased from Molecular Probes (Invitrogen, CA), fluorescein isothiocyanate (FITC) was purchased from Thermo Fisher Scientific, and 5-bromo-2′-deoxyuridine (BrdU) was purchased from Sigma-Aldrich.

### 2.4. Flow Cytometric Analysis

For FACS analysis, single-cell suspensions were made from lymph nodes (LN) and spleens of mice and cells were first incubated with appropriate monoclonal antibodies in staining buffer (Dulbecco's, 1% fetal bovine serum (FBS) and 0.1% sodium azide), washed twice, fixed in HBSS with 2% formalin, and then analyzed using FACSCalibur (BD Biosciences). For intracellular staining, cells were fixed and permeabilized following the manufacturer's instructions (eBiosciences). For CFSE labeling, cells were labeled in complete media with CFSE (10 *μ*M) for 15 min at 37°C and washed 3 times before use in experiments.

### 2.5. Cell Purification

For adoptive transfer experiments, lymphocytes harvested from the spleen and lymph nodes were processed in HBSS supplemented with 2% heat-inactivated FBS (HyClone, UT). CD4^+^CD25^−^ T cells were purified by first enriching for CD4 cells using T and CD4 affinity columns (R&D Systems, MN), followed by separation of CD4^+^CD25^−^ and CD4^+^CD25^+^ cells using PE anti-CD25 mAb and anti-PE magnetic beads (Miltenyi Biotec, CA), and then sorted by high-speed sorter (MoFlo, Cytomation, CO or FACSAria, BD Biosciences, CA). The purity of CD4^+^CD25^−^ or CD4^+^CD25^+^ cells was confirmed by FACS analysis before each transfer or *in vitro* experiment.

### 2.6. *In Vitro* T Cell Suppression Assay

CD4^+^CD25^+^ cells and CD4^+^CD25^−^ responder T cells (10000 cells per well) were purified as described above and then cultured in a 96-well round-bottomed plate at the indicated ratio with irradiated spleen cells (1 × 10^5^ cells) as APCs and soluble anti-CD3 antibody (0.5 *μ*g/ml) for 3 days at 37°C in 5% CO_2_. H^3^-thymidine (0.5 *μ*Ci) was added for the last 18 hrs, and H^3^-thymidine uptake was measured by a scintillation counter.

### 2.7. Adoptive Transfers

Purified congenic or CSFE-labeled donor CD4^+^CD25^−^ or CD4^+^CD25^+^ cells were suspended in 200 *μ*l of HBSS and injected intravenously into recipient mice. Peripheral lymph nodes and spleens were collected at various times after donor cell injection and cells were analyzed by FACS. Donor cells were detected in recipients by CFSE, CD45.1, CD45.2, or Thy1.2 labeling. All transferred populations were evaluated for Foxp3 expression by FACS prior to transfer, and the percentage of Foxp3^+/−^ cells taken into account in the calculations is described below. The calculation of percent recovery of donor cells was based on the following: (a) the absolute number of transferred donor cell populations and (b) the absolute number of donor cell populations in peripheral lymphoid organs (including both the lymph nodes and spleen) in the recipients.

### 2.8. *In Vivo* BrdU Labeling

Mice were injected intraperitoneally with 1.0 mg BrdU in 200 *μ*l PBS every 12 hours for three consecutive days (days -3, -2, and -1). On days 0, 5, 10, or 20 after injection, lymph node and spleen cells were collected from each mouse and assessed for intracellular BrdU incorporation as described below.

### 2.9. Intracellular Staining for BrdU and Foxp3

BrdU injection was performed as described above. Cells were surface stained with CD4 and CD25 antibodies, washed, and then fixed for 5 minutes at 37°C in 4% formaldehyde. Cell suspensions were then permeabilized by incubating for one hour on ice in PBS containing 0.1% Triton X-100. Permeabilized cells were incubated for 10 min at 37°C in RPMI 1640 containing recombinant DNase. Cells were then washed and incubated on ice for 40 minutes with FITC-labeled anti-BrdU antibody following the manufacturer's protocol. Suspensions were washed and then incubated overnight with anti-FoxP3 antibody in PBS containing 0.5% BSA and analyzed by flow cytometry.

### 2.10. FITC Labeling of Thymocytes

Details of this technique have been described previously [29, 30]. Briefly, animals were anesthetized and the chest was opened to expose the thymic lobes. Each thymic lobe was injected with 10 *μ*l of 350 *μ*g/ml fluorescein isothiocyanate (FITC) in PBS. After 24 hours, lymphoid organs (LN, spleen, and thymus) were removed for analysis. The FITC-injected thymus was always removed last to avoid cross-contamination of samples. Instruments were washed after removal of each organ. Recent thymic emigrants (RTE) in lymphoid organs were identified as live-gated FITC^+^ cells. This generally results in random labeling of 20–40% of the thymocyte populations.

### 2.11. Calculation of the Daily Thymic Export Rate

Details of this method have been described previously [29, 30]. The calculation of the daily thymic export rate was based on the following: (a) the percentage of thymocytes that are FITC^+^, (b) total cell counts for the thymus, spleen, and lymph nodes (pooled from inguinal, axillary, brachial, and cervical LN), (c) the proportion of T cells within these organs that were FITC^+^, thereby indicating export from the FITC-injected thymus, and (d) the time between FITC injection and harvest (24 hours after FITC injection). The daily CD4^+^*Foxp3^+^/Foxp3^−^* cell export rate was calculated using the following formula: daily CD4^+^CD8^−^Foxp3^+^ (or Foxp3^−^) cell export rate = absolute number of FITC^+^CD4^+^CD8^−^Foxp3^+^ (or Foxp3^−^) cells in peripheral pool/absolute number of FITC^+^CD4^+^CD8^−^Foxp3^+^ (or Foxp3^−^) cells in thymus + peripheral pool.

This calculation represents an estimate of the total number of cells exported from the injected thymus in the previous 24 hours. The peripheral pool was estimated as the total number of spleen cells plus twice the total number of lymph node cells.

### 2.12. Statistical Analyses

Data were analyzed by either Student's *t*-test or ANOVA and the Tukey-Kramer multiple comparisons test. Representative graphs of at least two independent experiments with consistent results are shown.

## 3. Results

### 3.1. Decreased Percentages of CD4^+^Foxp3^+^ Tregs in NOD Compared to B6 Mice Are First Observed Shortly before the Development of Full-Blown Diabetes

**Figure 1:**
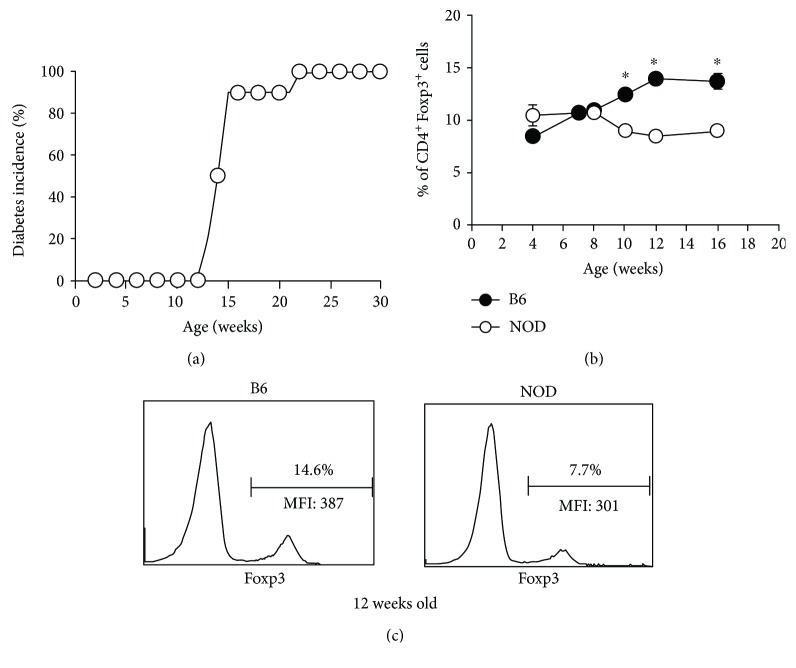
Low percentages of CD4^+^Foxp3^+^ cells are found in the periphery of NOD mice shortly before onset of overt diabetes. (a) Female NOD mice were monitored weekly for glycosuria and considered diabetic when they had two consecutive positive readings (>250 mg/dl). Diabetes incidence is expressed as a percentage of the mice that are positive for glucose (*n* = 10). (b) Percentages of CD4^+^ cells that express Foxp3 were analyzed in the peripheral lymph nodes of female NOD and B6 mice at varying ages. ∗ denotes a significant difference at *p* < 0.05 (*n* = 3-10). (c) Sample histograms of Foxp3 expression in CD4^+^ cells from 12-week-old female B6 and NOD mice.

NOD mice develop type 1 diabetes (T1D) spontaneously and share several critical features with the human disease. Diabetes incidence and progression are highly dependent on the animal facility, and the incidence of T1D across facilities ranges from 60 to 100% [1, 12–15, 17–20]. This is probably due to differences in gut microbiota that are, subsequently, dependent on variations in food, water, and general environment [31, 32]. We monitored female NOD mice in our animal facility for diabetes onset and incidence. Mice were considered diabetic after two consecutive positive readings (>250 mg/dl) for glycosuria, and incidence is expressed as a percentage of total mice monitored that develop disease. Female NOD mice in our facility begin developing full-blown T1D by about 13-15 weeks of age and the incidence of T1D in our colony is consistently 90-100% by 23 weeks of age (Figure 1(a)). Similar to discrepancies observed in the age of onset and disease incidence in different colonies of NOD mice, the differences in the percentages of CD4 Tregs in NOD vs control mice also vary between animal facilities. Some laboratories, including ours, have reported observing lower percentages of CD4^+^ Tregs in NOD mice in the periphery by comparison to autoimmune-resistant mice [1, 8, 10] whereas others report no differences [2, 3, 6]. Most of the earlier studies were limited to using CD25 as a marker for Tregs because reagents that could detect Foxp3 directly by flow cytometry were not commercially available at that time. In the present study, we analyzed the levels of Tregs in LN of NOD mice at different ages before and after diabetes onset in our colony using Foxp3 as a marker. We found that there were no differences in the percentages of CD4^+^Foxp3^+^ cells (i.e., the percentages of CD4^+^ cells that are Foxp3^+^) in young (4-8 wk old prediabetic) NOD mice compared to the control B6 strain (Figure 1(b)). In contrast, at ~9-10 weeks of age (i.e., shortly before diabetes onset), the percentages of CD4^+^Foxp3^+^ (Figure 1(b)) cells were significantly lower in NOD mice than B6 controls. Furthermore, unlike B6 mice, NOD mice exhibited percentages of CD4^+^Foxp3^+^ cells that either decreased or did not increase with age and remained lower than B6 mice through at least 16 weeks of age (Figures 1(b) and 1(c)). Therefore, a relatively low Treg to effector CD4 cell ratio in NOD, compared to B6, mice is first observed shortly before the development of full-blown diabetes in our colony. These data confirm our previous studies showing that the percentages of CD4^+^CD25^+^ cells in adult female NOD mice in our facility are significantly lower than those in B6 mice [1].

### 3.2. No Differences in Treg Function between NOD and B6 Mice Using an In Vitro Suppression Assay

**Figure 2:**
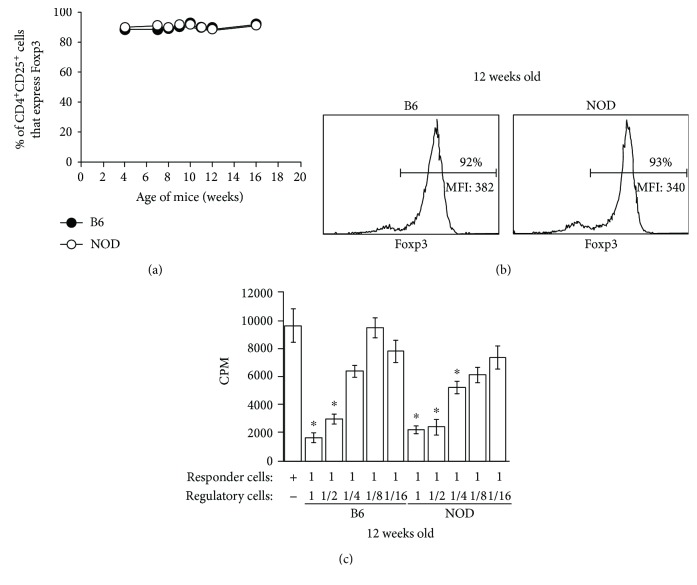
NOD and B6 CD4^+^ Tregs exhibit equivalent suppressive activity *in vitro*. (a) Percentages of CD4^+^CD25^+^ cells that express Foxp3^+^ in peripheral lymph nodes of female NOD and B6 mice at varying ages (*n* = 3-10). (b) Sample histograms of Foxp3 expression in CD4^+^CD25^+^ cells from peripheral lymph nodes of 12-week-old female B6 and NOD mice. (c) Lymphoid cells from NOD and B6 mice were collected at 12 weeks of age and CD4^+^CD25^+^ cells sorted. Varying numbers of CD4^+^CD25^+^ cells were cocultured with age-matched NOD CD4^+^CD25^−^ responder cells, irradiated splenocytes, and anti-CD3 *in vitro*. Proliferation was determined by detection of H^3^-thymidine uptake. ∗ denotes a significant difference from positive control at *p* < 0.01.

Some, but not all, studies have suggested a progressive waning in CD4^+^ Treg function as a triggering mechanism for T1D onset and progression [2, 3, 6, 11]. We have found that the CD4^+^CD25^+^ population in NOD and B6 mice at 4-16 weeks of age contains very similar percentages (>90%) of Foxp3^+^ cells (Figures 2(a) and 2(b)). Furthermore, although we observed that Tregs in NOD mice in our colony exhibit no differences in Foxp3 expression (MFI) at 4 weeks of age (B6: 365 ± 23 vs NOD: 374 ± 35), Tregs from NOD mice at 12 weeks of age did express slightly lower levels of Foxp3 compared to those from B6 mice (B6: 372 ± 13 vs NOD: 294 ± 47; Figure 2(b)). To evaluate inherent Treg function *in vitro*, we performed the classic proliferation suppression assay, comparing NOD and B6 Tregs from mice at different ages. For these experiments, varying numbers of purified CD4^+^CD25^+^ cells from 4- to 12-week-old female NOD or B6 mice were cocultured with age-matched CD4^+^CD25^−^ cell responders from either NOD or B6 (data not shown) mice and anti-CD3. As shown in Figure 2(c), we found that NOD CD4^+^CD25^+^ cells from 12-week-old NOD mice exhibited suppressive function in a standard *in vitro* assay that was comparable to B6 CD4^+^CD25^+^ cells. Similar results were found at 3, 4, 6, and 10 weeks of age (data not shown). Our data suggest that although there is a quantitative decrease in the Treg to Teff cell ratio (i.e., percentages of CD4^+^ that are Foxp3^+^) by 9-10 weeks of age in NOD mice in our colony, the Tregs from NOD mice do not exhibit a functional defect in the *in vitro* assay compared to Tregs from B6 mice.

### 3.3. No Differences in Survival/Persistence of Peripheral CD4^+^ Tregs between NOD and B6 Mice

**Figure 3:**
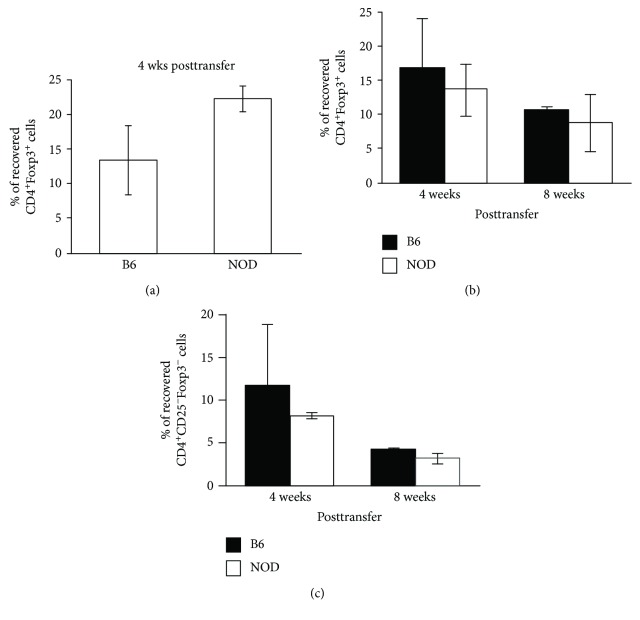
Transferred NOD CD4^+^ Tregs persist/survive similarly to B6 controls under both lymphopenic and nonlymphopenic conditions. (a) Five hundred thousand purified CD4^+^CD25^+^ cells (~92% Foxp3^+^) from NOD or B6 mice were transferred into NOD.RAG or B6.RAG (lymphopenic) recipients, respectively. Four weeks after transfer, LN and spleen cells were collected and absolute numbers were counted from individual samples. Percentages of CD4^+^ cells that express Foxp3 were analyzed. The percent recovery of transferred cells was calculated based on the absolute number of recovered donor CD4^+^Foxp3^+^ cells divided by the total cell number injected. (b, c) Five hundred thousand purified CD4^+^CD25^+^ cells and CD4^+^CD25^−^ cells from NOD (Thy1.2) or B6.SJL (CD45.1) mice were transferred into NOD.NON (Thy1.1) or B6 (CD45.2) intact (nonlymphopenic) recipients, respectively. Four or 8 weeks after transfer, LN and spleen cells were collected and the absolute number of cells from individual samples were counted. Percentages of CD4^+^ cells that did or did not express Foxp3 were analyzed. The percent recovery of transferred cells was calculated based on the absolute number of recovered donor CD4+Foxp3^+^ (b) or CD4+CD25^−^Foxp3^−^ (c) cells divided by the total number of respective donor cells injected × 100 (*n* = 5).

Several mechanisms that contribute to the maintenance of both Treg and Teff populations in the periphery may influence the balance between these two populations (i.e., the Treg to T effector cell ratio), including persistence and/or survival, peripheral homeostatic T cell proliferation/expansion, and thymic production and output of each population. It has been reported that human CD4^+^CD25^+^ regulatory T cells are highly susceptible to spontaneous cell death or cytokine deprivation-induced cell death [33]. In the following experiments, we first compared the persistence of NOD and B6 Tregs transferred into a lymphopenic recipient where the transferred cells do not need to compete with endogenous cells for resources. This allowed us to determine if there were any inherent defects in the ability of NOD Tregs to persist/survive *in vivo*. For these experiments, purified CD4^+^CD25^+^ cells (transferred population is ~92% Foxp3^+^) from NOD or B6 mice were transferred into NOD.RAG or B6.RAG recipients, respectively. Four weeks after transfer, lymphoid organs were collected and the percentages of donor CD4^+^Foxp3^+^ cells that were recovered were determined (# Foxp3^+^ cells transferred/# Foxp3^+^ cells recovered). There were no significant differences in the relative percentages of recovered CD4^+^Foxp3^+^ cells between NOD and B6 recipients (Figure 3(a)), and we, therefore, concluded that NOD CD4^+^Foxp3^+^ cells exhibited no inherent defect in their ability to persist or survive *in vivo* for an extended period of time (i.e., 4 weeks), at least under conditions where they do not need to compete for resources.

Next, we assessed the long-term persistence of CD4^+^ Tregs or Teff cells in NOD mice under normal nonlymphopenic conditions where the cells do, in fact, need to compete with endogenous cells for resources. For these experiments, NOD (Thy1.2^+^) CD4^+^CD25^+^ cells or CD4^+^CD25^−^ cells were transferred into intact congenic recipient NOD.NON (Thy1.1^+^) mice. Congenic B6.SJL (CD45.1^+^) CD4^+^CD25^+^ or CD4^+^CD25^−^ cells transferred into B6 (CD45.2^+^) recipients served as controls. Four or 8 weeks after transfer, lymphoid organs were collected and recovered donor cells were analyzed. No differences were found in the maintenance or persistence (i.e., the percent of donor cells recovered) of either CD4^+^Foxp3^+^ or CD4^+^CD25^−^Foxp3^−^ cells (Figures 3(b) and 3(c)) between NOD and B6 mice. These data indicate that both NOD CD4^+^Foxp3^+^ and CD4^+^Foxp3^−^ cells persist and appear to survive in a manner similar to B6 cells and further suggest that a difference in survival is not a likely mechanism that leads to differences in the percentages of CD4^+^Foxp3^+^ cells in the periphery of NOD mice.

**Figure 4:**
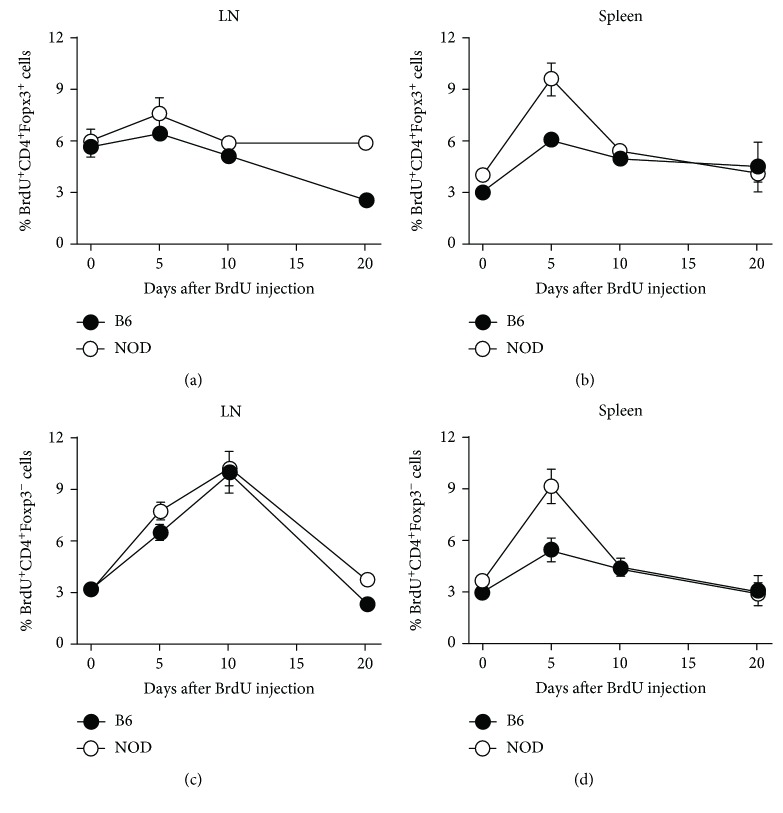
NOD CD4^+^Foxp3^+^ and CD4^+^Foxp3^−^ cells survive similarly to B6 control cells *in situ*. Seven-week-old NOD or B6 mice were injected with BrdU i.p. every 12 hours for 3 days (days –3, -2, and -1). On days 0, 5, 10, and 20 postinjection, LN (a,c) and spleen (b, d) cells were collected and labeled with CD4, CD25, Foxp3, and BrdU antibodies. CD4^+^Foxp3^+^ (a, b) or CD4^+^Foxp3^−^ (c, d) cells in the LN and spleen were gated and the percentages of BrdU^+^ cells were determined (*n* = 3).

Although the experiments described above test for persistence and to a certain extent survival, they do not evaluate the survival capacity of the CD4^+^Foxp3^+^ cells *in situ* or rule out any effect of proliferation on persistence/survival. The following experiments were designed to determine how well endogenous CD4^+^Foxp3^+^ and CD4^+^Foxp3^−^ cells survive in intact NOD or B6 mice. Survival of CD4^+^Foxp3^+^ and CD4^+^Foxp3^−^ cells was evaluated *in vivo* by administering BrdU to intact NOD and B6 mice for 3 days (days -3, -2, and -1) then determining the retention of BrdU^+^CD4^+^ cells in secondary lymphoid organs at 0, 5, 10, or 20 days after the final BrdU injection. BrdU incorporates into the newly synthesized DNA of replicating cells (during the S phase of the cell cycle); then BrdU is used to track the cells over time. Only the BrdU^hi^ cells were analyzed to ensure that the cells that were evaluated had not undergone proliferation. These analyses were performed during the time frame that coincided with the decreased percentages of CD4^+^Foxp3^+^ cells in NOD mice, i.e., between 7 and 10 weeks of age (see Figure 1(b)). As shown in Figure 4, no differences in the persistence or survival of either CD4^+^Foxp3^+^ (Figures 4(a) and 4(b)) or CD4^+^Foxp3^−^ (Figures 4(c) and 4(d)) cells that could account for the lower Treg (Foxp3^+^) to T eff (Foxp3^−^) cell ratios between NOD and B6 mice were observed in either the LN (Figures 4(a) and 4(c)) or the spleen (Figures 4(b) and 4(d)) at the different time points after BrdU injection. This experimental design allowed us to evaluate short-term survival of these endogenous T cell populations.

### 3.4. No Differences in Peripheral CD4^+^ Treg Homeostatic Proliferation/Expansion in NOD and B6 Mice at either Prediabetes or Peridiabetes Time Points

The number of T cells that are maintained in the periphery after exportation from the thymus is dependent on homeostatic proliferation/expansion, survival/persistence, and death [2, 29, 33, 34]. Dysregulation of any of these mechanisms can alter T cell populations and Treg-to-T effector cell ratios and contribute to pathogenic T cell generation or loss of tolerance. In the following experiments, we compared the ability of CD4^+^CD25^+^ Tregs from NOD and B6 mice to undergo homeostatic proliferation under either lymphopenic or nonlymphopenic conditions.

**Figure 5:**
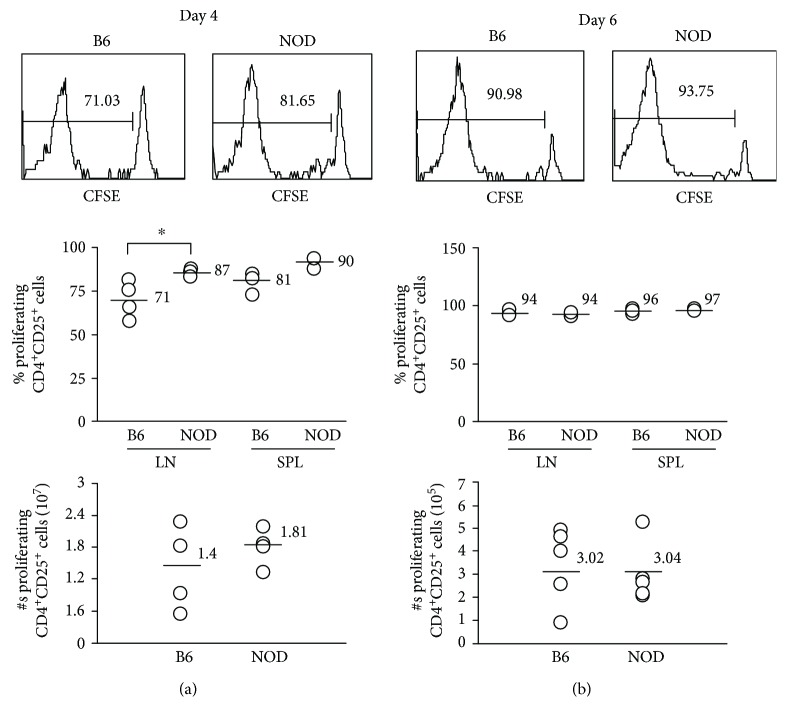
Transferred NOD and B6 CD4^+^ Tregs undergo comparable homeostatic proliferation in lymphopenic mice. Five hundred thousand purified CD4^+^CD25^+^ cells from NOD or B6 mice were labeled with CFSE and transferred into NOD.SCID or B6.SCID mice, respectively. (a) Four or (b) six days after transfer, LN and spleen cells were collected and labeled with CD4 and CD25 antibodies. CD4^+^ cells were gated and analyzed for CFSE dilution among donor cells. Percentages (a and b, upper and center panels) and absolute numbers (a and b, lower panels) of CFSE low (i.e., cells that have undergone proliferation) donor cells are shown. Each symbol represents data from an individual animal.

We, first, analyzed homeostatic proliferation of donor CD4^+^ cells transferred into lymphopenic recipients. For these experiments, CD4^+^CD25^+^ cells from NOD or B6.SJL mice were CFSE labeled and transferred into syngeneic NOD.SCID or B6.SCID recipients, respectively. Proliferation of CFSE-labeled transferred cells was assessed in the spleen and LN on days 4 and 6 following transfer (Figures 5(a) and 5(b), respectively). Although no differences were found in the ability of NOD or B6 CD4^+^CD25^+^ cells to undergo homeostatic proliferation at either time point in the spleen or at 6 days in the LN, there was a slight increase in the percentage of proliferating (CFSE^lo^) CD4^+^CD25^+^ cells recovered from the NOD LN on day 4 compared to B6 (Figure 5(a) top and center panels). However, there were no differences in the total numbers of proliferating cells at either 4 or 6 days after transfer (Figures 5(a) and 5(b), bottom panels). These data suggest that NOD CD4^+^CD25^+^ cells do not have inherent defects in their ability to undergo homeostatic proliferation under lymphopenic conditions.

**Figure 6:**
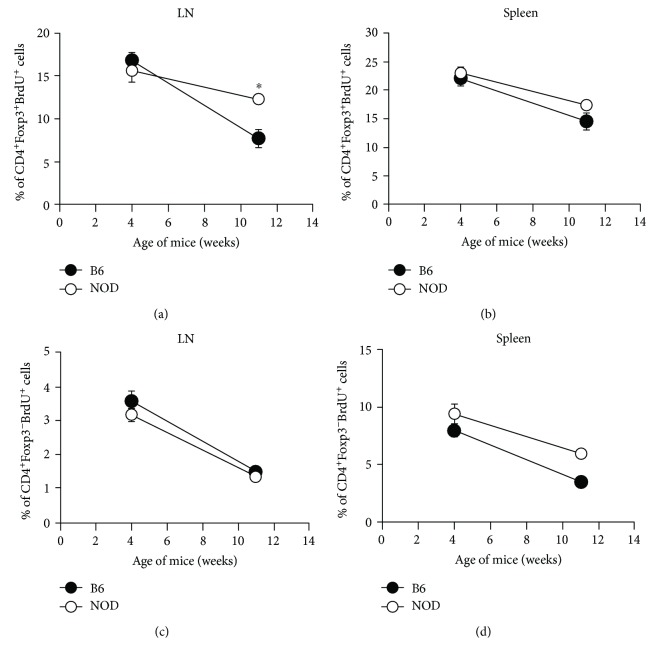
Differences in NOD vs B6 CD4^+^ Treg and non-Treg homeostatic proliferation do not account for the decrease in Treg percentages in the periphery of NOD mice. Four- or 11-week-old NOD or B6 mice were injected i.p. with BrdU every 12 hours for 3 days (days –3, -2, and –1). On day 0 (i.e., 12 hours after final BrdU injection), LN (a, c) and spleen (b, d) cells were collected and labeled with antibodies for CD4, Foxp3, and BrdU. CD4^+^Foxp3^+^ (a, b) or CD4^+^Foxp3^−^ (c, d) cells were gated and analyzed for BrdU incorporation (*n* = 5).

Under nonlymphopenic conditions (i.e., in an intact wild-type mouse) where T cells must compete with other resident lymphoid cells (e.g., other T cells) for growth factors and other resources, it is possible that NOD CD4^+^ Tregs may be less robust and therefore unable to expand. To test this, we compared the proliferation of endogenous CD4^+^Foxp3^+^ and CD4^+^Foxp3^−^ cells in NOD and B6 mice *in vivo* at 4 and 11 weeks of age by injecting mice with BrdU to label the DNA of dividing cells. NOD or B6 mice were injected with BrdU every 12 hours for 3 days, and cells were harvested 12 hours after final BrdU injection. Percentages of CD4^+^Foxp3^+^ and CD4^+^Foxp3^−^ cells that had incorporated BrdU (and had, therefore, proliferated) were compared by flow cytometry. CD4^+^Foxp3^+^ and CD4^+^Foxp3^−^ cells from four-week-old NOD and B6 mice exhibited comparable BrdU incorporation in both LN and spleen, i.e., exhibited similar levels of proliferation (Figure 6). However, CD4^+^Foxp3^+^, but not CD4^+^Foxp3^−^, cells in the LN from 11-week-old NOD mice exhibited slightly greater incorporation of BrdU, i.e., proliferated slightly more, compared to B6 mice (Figure 6(a)). There were no significant differences in proliferation in the spleen for either CD4^+^Foxp3^+^ or CD4^+^Foxp3^−^ cells at either 4 or 11 weeks of age (Figures 6(b) and 6(d)). Taken together, these data suggest that differences in proliferation of either the CD4^+^Foxp3^+^ or CD4^+^Foxp3^−^ populations in peripheral lymphoid organs do not contribute to the lower percentages of CD4^+^Foxp3^+^ in NOD mice.

### 3.5. NOD Mice Do Not Produce Lower Levels of CD4^+^Foxp3^+^ Cells in the Thymus Compared to B6 Mice

**Figure 7:**
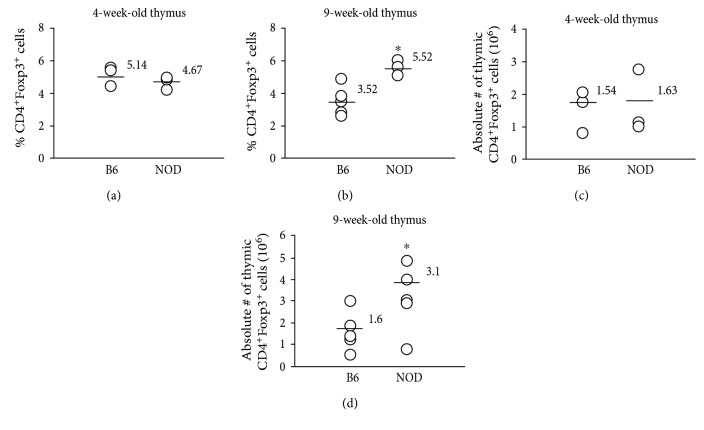
Percentages and absolute numbers of CD4^+^Foxp3^+^ cells are not decreased in the thymus in NOD compared to B6 mice at either 4 or 9 weeks of age. Thymocytes were collected from NOD or B6 mice at (a, c) 4 or (b, d) 9 weeks of age and evaluated for the percentages (a, b) and absolute numbers (c, d) of CD4^+^Foxp3^+^ cells. Each symbol represents data from an individual animal. ∗ denotes a significant difference at *p* < 0.05.

The thymus serves as the primary source of naturally occurring Foxp3-expressing cells. Defects in the thymus of NOD mice are well documented and include abnormal thymic selection of T cells and possibly abnormal Tregs [35–37]. Defects in the ability of NOD mice to produce CD4^+^Foxp3^+^ cells in the thymus could have a direct impact on the frequency of CD4^+^Foxp3^+^ cells in the periphery. To determine whether the differences in the percentages of peripheral Tregs between NOD and B6 mice are caused by differences in thymic production of this population, the thymi from 4- and 9-week-old NOD or B6 mice were analyzed for the percentages and absolute numbers of CD4^+^Foxp3^+^ cells. As shown in Figure 7, neither the percentages nor the absolute numbers of CD4^+^ cells expressing Foxp3 in the thymus of NOD mice were decreased at either 4 or 9 weeks of age when compared to B6 mice. In fact, the opposite was actually true, a greater percentage of thymic CD4^+^ cells expressed Foxp3, and the absolute numbers of CD4^+^Foxp3^+^ cells were greater in 9-week-old NOD compared to B6 mice (Figures 7(b) and 7(d), respectively). Therefore, the lower percentages of peripheral CD4^+^Foxp3^+^ cells in NOD mice are not reflected in a proportional decrease in their percentage or number in the thymus, suggesting that there is not a defect in the production of CD4^+^Foxp3^+^ cells in the thymus of NOD mice.

### 3.6. There Are No Differences in Thymic Output of CD4^+^Foxp3^+^ Cells, but the Thymic Output of CD4^+^Foxp3^−^ Cells Is Much Greater in NOD Mice

**Figure 8:**
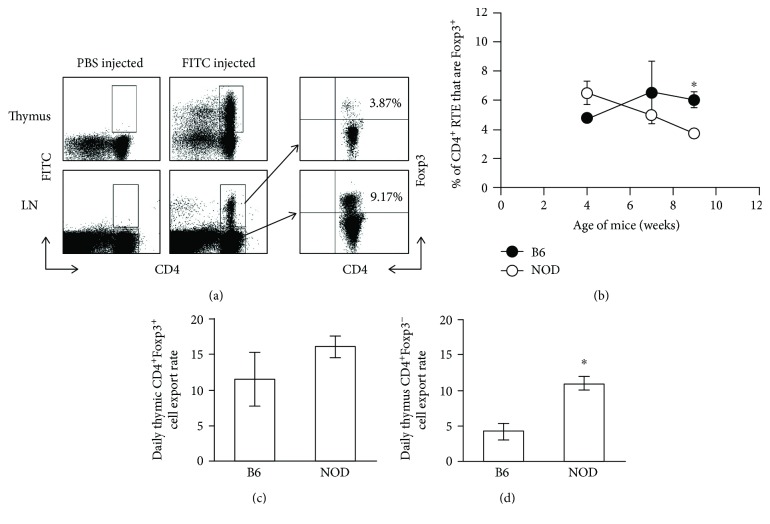
Percentages of CD4^+^ recent thymus emigrants (RTE) that are Foxp3^+^ are lower in NOD compared to B6 mice at 9 weeks of age and may be due to the higher rate of thymic output of CD4^+^Foxp3^−^ cells in NOD mice. Both thymic lobes of NOD and B6 mice were injected with 10 *μ*l of either (a) PBS (upper left panel) or FITC (upper center panel) at 4, 7, or (a) 9 weeks of age. This typically resulted in random labeling of 20-40% of the thymocyte population. Mice were sacrificed 24 hours after injection, and lymphoid organs (LN and spleen) were collected. FITC^+^CD4^+^ cells (RTE) in secondary lymphoid organs were gated (a, lower center panel) and analyzed for the percentages of Foxp3^+^ (a, upper panel right, and b). (c, d) The daily (24 h) thymic export rates of CD4^+^Foxp3^+^ (c) or CD4^+^Foxp3^−^ (d) cells in 9-week-old mice were determined using the formula described in Methods. ∗ denotes a significant difference at *p* < 0.01.

Although thymic production of CD4^+^Foxp3^+^ cells (i.e., percentages and absolute numbers of CD4^+^Foxp3^+^ cells in the thymus; see Figure 7) may not be lower in NOD mice, the increased percentage and number of these cells in NOD thymi at 9 weeks of age (see Figures 7(b) and 7(d)) suggested that there may be a defect in the ability of these cells to emigrate to the periphery. Using a classic cell tracking technique in which FITC is injected into the thymus [2, 30], we were able to quantify and compare the rate of thymic output of CD4^+^Foxp3^+^ cells in NOD and B6 mice. Intrathymic injection of FITC results in random labeling of thymocytes (at ~20-40% labeling efficiency; Figure 8(a), upper center panel), but not cells in the periphery, and therefore, only recent thymic emigrants (RTE) will be FITC^+^ in the periphery [2, 30]. For these experiments, FITC was injected directly into each lobe of the thymus of 4, 7, or 9 wk old mice and lymphoid organs were collected after 24 hours. As shown in Figure 8(a) (lower center panel), FITC^+^CD4^+^ cells could be detected in the periphery (i.e., RTE; the majority of peripheral FITC^+^ cells were CD3^+^ (data not shown)). To determine the percentage of CD4^+^ RTE that were Foxp3^+^, FITC^+^CD4^+^ LN and spleen cells were gated and analyzed for the percentage of Foxp3^+^ cells (Figure 8(a) upper right panel). No differences were found in the percentages of CD4^+^ RTE that are Foxp3^+^ in NOD mice compared with normal B6 controls at 4 and 7 weeks of age (Figure 8(b)). However, significant decreases in this population were found at 9 weeks of age (Figure 8(b)), i.e., at approximately the age that lower percentages of CD4^+^Foxp3^+^ cells are found in the periphery in NOD mice (see Figure 1(b)), and shortly before the mice begin to develop full-blown diabetes (see Figure 1(a)). These data suggest that the thymic CD4^+^Foxp3^+^ cell export rate may be lower in NOD mice. To test this, the daily CD4^+^Foxp3^+^ thymic export rate was calculated using total numbers of FITC^+^CD4^+^Foxp3^+^ cells in the thymus, LN, and spleen in the formula described in Methods. No differences were found in the rate of thymic output of CD4^+^Foxp3^+^ in NOD mice compared to B6 mice at either 4 or 7 weeks of age (data not shown) or, contrary to our hypothesis, at 9 weeks of age (Figure 8(c)). Taken together, these data indicate that the lower levels of CD4^+^Foxp3^+^ Tregs found in the periphery of NOD mice are not due to deficiencies in their production (see Figure 7) and may not be due to defects in their release by/emigration from the NOD thymus (Figure 8(c)).

Since either a decreased output of CD4^+^Foxp3^+^ cells or increased output of CD4^+^Foxp3^−^ cells could change the balance of CD4^+^Foxp3^+^ cells to CD4^+^Foxp3^−^ cells in the periphery and we had already ruled out the former, an alternative possibility is that the decreased percentage of CD4^+^Foxp3^+^ RTE that are found in NOD mice at 9 weeks of age is due to an increase in the daily thymic export rate of CD4^+^Foxp3^−^ T cells in NOD mice. To investigate this possibility, we calculated the daily rate of the CD4^+^Foxp3^−^ thymic output. In calculating the daily T cell export rate, we found that 9-, but not 4- or 7- (data not shown), week-old NOD thymus glands exported significantly more CD4^+^Foxp3^−^ T cells than B6 mice (Figure 8(d)). These data suggest that the lower percentages of CD4^+^Foxp3^+^ cells in the periphery of NOD mice may be, at least in part, due to an increase in the output of CD4^+^Foxp3^−^ cells by the thymus relative to the output of CD4^+^Foxp3^+^ cells.

## 4. Discussion

Although the incidence of type 1 diabetes is increasing in industrialized countries, there are no effective treatments to halt the progression of autoimmunity largely because the mechanisms underlying disease pathogenesis remain incompletely understood. The NOD mouse model is an invaluable tool for the study of the autoimmune mechanisms at play in T1D because of its remarkable similarity to human disease from both genetic and pathogenesis standpoints. Compelling evidence supports an important role for Tregs in keeping potential pathogenic T cells in check and regulating autoimmune diseases, including T1D. In this paper, we show that the higher thymic output of CD4^+^Foxp3^−^ cells may contribute to the imbalance between pathogenic T cells and Tregs that is reflected in the low percentages of peripheral CD4^+^Foxp3^+^ cells in NOD mice in our animal facility.

Several lines of investigation support the hypothesis that destruction of self-tissue is strictly controlled by adequate numbers of functional Tregs and an effective Treg: effector CD4^+^ cell balance. First, spontaneous diabetes incidence is increased to 100% and disease onset is accelerated in NOD mice deficient in either CD28 or CD80 and CD86 that is directly correlated with a quantitative decrease in Tregs by >80% [8]. Similarly, Treg depletion early in life by treatment with IL-2-neutralizing antibody results in early diabetes onset [38]. Furthermore, therapies that augment Treg numbers have beneficial effects on the development of diabetes [21, 23–25].

The incidence of T1D in NOD mice in our facility is high and consistently ranges from 90 to 100% (see Figure 1(a)) [1, 12]. T1D susceptibility is not only under polygenic control (i.e., associated in both mice and humans with >20 different genes), but is also strongly influenced by the environment in mice and probably in humans as well. For example, the differences in T1D incidence in NOD mice in different animal facilities are likely due to the degree of cleanliness and exposure to microbial and dietary factors that can generate distinct commensal microbiota and associated metabolites and influence disease onset. Paradoxically, the cleaner the facility, the higher the frequency and the earlier the onset of type 1 diabetes in NOD mice. The incidence and age at disease onset in NOD mice are highly correlated with the cleanliness of animal facilities, and incidence usually ranges from 60 to 80%. It is now well established that the incidence of T1D in NOD mice is influenced by gut microbiota as well as dietary factors [13–15, 17–20]. Mice bred and maintained under specific pathogen-free (SPF) conditions are more predisposed to the development of diabetes compared to those bred under conventional conditions [14]. Furthermore, administration of probiotics can prevent diabetes in NOD mice and spontaneous disease-prone BioBreeding rats (BB-DP) housed under SPF conditions [20, 39–41]. Interestingly, evidence suggests that the pH of drinking water may influence the composition of gut microbiota and, consequently, the incidence of type 1 diabetes in NOD mice [31]. Finally, in human patients, a correlation between the diversity of the intestinal microbiota and the occurrence of T1D or anti-islet cell autoimmunity has been reported [42–45]. Overall, accumulating evidence suggests that a variety of environmental factors can have a significant impact on autoimmune diseases such as type 1 diabetes and differences in environmental factors can explain the variation in disease incidence in NOD mice housed in different animal facilities.

The percentages of CD4^+^CD25^+^ Tregs in NOD mice also appear to vary markedly between facilities, with some laboratories reporting no differences in percentages between NOD and control strains such as B6 mice [2, 3, 6] and others finding significant differences [1, 8, 10]. We report here that the percentages of CD4^+^Foxp3^+^ cell populations in the periphery of NOD mice in our animal facility are significantly lower in comparison to non-autoimmune-prone B6 mice beginning at about 10 weeks of age, i.e., shortly before high blood glucose is first detected in our facility at ~12 weeks of age (these mice would be considered peridiabetic; mice develop full-blown diabetes at ~13-15 weeks in our facility). Interestingly, unlike B6 mice, the relative percentages of CD4^+^Foxp3^+^ cells did not increase with age and remained relatively constant from 4 to 16 weeks of age whereas the percentages of CD4^+^Foxp3^+^ cells in B6 mice steadily increased during the same time period. On the other hand, we found no differences in function between NOD or B6 Tregs at any age using the classic *in vitro* proliferation assay. This was true whether the T cell responders were from age-matched NOD (we did not test mice older than 12 weeks of age) or B6 mice. Taken together, these data indicate that relatively low Treg frequencies, but not a defect in Treg function, correlate with disease onset in our animal facility.

Alterations in Treg frequencies (i.e., the Treg : T effector ratio) can be due to either a decrease in the Treg population and/or a relative increase in the nonregulatory or Teff cell population. T cell populations, both Tregs and T effector cells, are maintained in the periphery through homeostatic proliferation, persistence and/or survival, and thymic production and output of each population. In the current study, we evaluated each of these mechanisms to determine whether one or more contributed to the low Treg frequencies in NOD mice in our facility. Thymic defects in NOD mice are well documented, and in particular, evidence suggests that negative selection is defective in NOD mice [37]. On the other hand, thymic selection of Foxp3^+^ Tregs in NOD mice is not defective, although NOD Tregs do have reduced TCR diversity [35, 36]. We evaluated thymic production of Tregs (i.e., CD4^+^Foxp3^+^ cells) in NOD mice in our facility and determined the percentages and absolute numbers of CD4^+^Foxp3^+^ cells in the thymus of NOD and B6 mice. We found that NOD mice did not have decreases in percentages or numbers of CD4^+^Foxp3^+^ cells in the thymus at either 4 or 9 weeks of age, and in fact, NOD mice had even higher percentages and numbers at 9 weeks of age than B6 mice, as has been reported previously [48]. These data indicated that NOD mice in our facility, as shown in other studies [35, 36, 46], do not appear to have a defect in their ability to produce CD4^+^Foxp3^+^ cells in the thymus. In fact, at least one study has suggested that selection of Foxp3^+^ Tregs may actually be enhanced in NOD mice [47] and this could account for the increased Foxp3^+^ cells that we find in the NOD thymus. However, the higher percentages and numbers in the NOD thymus at 9 weeks of age could also indicate that there might be an issue with the ability of the CD4^+^Foxp3^+^ cells to migrate out of the thymus in NOD mice as suggested by the study of Mendes-da-Cruz et al. [48] that shows that NOD thymocytes exhibit altered expression of CXCR4 and VLA-6, and CD4^+^Foxp3^+^ cells, in particular, have decreased expression of VLA-5 and altered trafficking capability in *in vitro* assays. This group further found that the NOD thymus also had increased levels of fibronectin, laminin, and CXCL12 deposition. Taken together, these data correlated with increased accumulation of thymocytes and CD4^+^Foxp3^+^ cells, in particular, in the perivascular spaces, and suggested that the CD4^+^Foxp3^+^ cells may have a defect in their ability to traffic out of the thymus, although that study did not directly evaluate the thymic output of CD4^+^Foxp3^+^ cells *in vivo* to confirm this possibility. We did, in fact, find that the percentages of CD4^+^ cells that were Foxp3^+^ among recent thymic emigrants (RTE) were significantly lower in NOD compared to B6 mice by 9 weeks of age. This result is contrary to an earlier study that reported no differences in RTE of CD4^+^CD25^+^ Tregs in NOD mice compared to diabetes-resistant strains [2] and could be due to the use of different markers to identify Tregs (i.e., CD25 vs Foxp3) in the two studies and/or the impact of the environment in different animal facilities as described below. Upon further analysis of our data, we discovered that the low Treg to effector T cell ratio in the NOD RTE in our study was likely due, not to a decrease in Treg output, but to an increase in thymic output of CD4^+^Foxp3^−^ cells. We believe, therefore, that it is likely that it is primarily through this mechanism that the imbalance between Tregs and effector T cells occurs in NOD mice. However, thymic T cell emigration is likely to be highly complex and the sequestration of Tregs in the thymus could also play a role in this Treg : T effector imbalance in the periphery and the accumulation of Tregs in the thymus in NOD mice as suggested previously [48] and there is a possibility that the *in vivo* thymic output assays cannot detect subtle differences in thymocyte-trafficking capabilities.

One possible reason for the differences in all of the studies described above is that facility-dependent microbiota may exert effects on lymphoid organs distant from the intestine. This is supported by a recent study suggesting that commensal bacteria can influence AIRE expression in thymic epithelial cells and may, therefore, potentially affect thymic selection [49]. The relationship between animal facilities and differences in type 1 diabetes incidence/age of onset has been well documented as described above, and although the underlying mechanisms remain unknown, based on the data presented here, we propose that Treg frequencies and thymic negative selection may play a role and may be influenced by alterations in the gut microbiota in animal facilities and possibly in human populations. This potential causal relationship requires further study.

## Data Availability

The data used to support the findings of this study are included within the article.
